# Is Learning With Elaborative Interrogation Less Desirable When Learners Are Depleted?

**DOI:** 10.3389/fpsyg.2019.00707

**Published:** 2019-03-29

**Authors:** Tim Kühl, Alex Bertrams

**Affiliations:** ^1^Psychology of Education, University of Mannheim, Mannheim, Germany; ^2^Educational Psychology, University of Bern, Bern, Switzerland

**Keywords:** desirable difficulties, elaborative interrogation, ego depletion, learning and instruction, multimedia learning, transfer

## Abstract

When learning with elaborative interrogation, learners are requested by means of prompts to generate parts of the study material. There is evidence, that learning with elaborative interrogation is beneficial. However, it is conceivable that for elaborative interrogation to be beneficial for learning, learners also need resources available to be able to correctly generate parts of the study material. In this connection, one potentially important factor for successfully carrying out such effortful analytic processes, like generating information, is cognitive self-control. However, self-control seems to be a limited resource that can be depleted. Hence, under conditions of depleted self-regulatory resources (ego depletion), elaborative interrogation might lead to an incomplete generation of the requested information, resulting in incomplete study material. Thus, elaborative interrogation may be only beneficial under nondepleted conditions, but disadvantageous under depleted conditions. To investigate this, 97 persons were randomly assigned to one of four conditions resulting from a 2 × 2 between-subjects design with the independent variables ego depletion (yes vs. no) and learning condition (elaborative interrogation vs. control). Ego depletion was manipulated with a writing task: Participants were instructed to transcribe a text on a blank sheet, but only participants in the depletion condition were instructed to omit the letters *e* and *n* wherever they would normally appear in their writing. For the elaborative interrogation condition, some segments of the regular text were removed and prompts asking for that particular information were provided. For the control condition, the regular text was provided while no prompts were given. The main dependent variables were the learning outcome measures of a retention test and a transfer test. 2 × 2-ANCOVAs showed no effects of ego depletion, no effects of learning condition and no interaction between ego depletion and learning condition – neither for retention nor for transfer. The concept of ego depletion is recently discussed controversy and these results do contribute to the skeptical view that queries the impact of the concept of ego depletion – at least for cognitive tasks. Moreover, these results question whether elaborative interrogation are also desirable when assessing learning outcomes by means of retention and transfer tests.

## Introduction

Overall, and as emphasized in different theoretical frameworks of learning and instruction, there is a general agreement that learners need to be actively involved in the learning process for gaining a better understanding of the instructional material (e.g., Chi, 2009; Mayer, 2009; Sweller et al., 2011). Unfortunately, however, learners do not necessarily actively engage by putting effort in processing instructional material. Fortunately, there are several established or promising instructional strategies to overcome this shallow processing (for overviews see Bjork and Bjork, 2011; Roediger and Pyc, 2012; Dunlosky et al., 2013; Fiorella and Mayer, 2016). One global type of strategy is based on introducing challenges. Such instructional strategies that aim at a better understanding of a content by making initial learning more difficult for a learner, can be subsumed to the framework of desirable difficulties (cf. Bjork, 1994; Bjork and Bjork, 2011). To this framework belong conditions where parts of the information have to be generated by a learner (Bjork and Bjork, 2011). This is the case for an instructional intervention called elaborative interrogation (cf. McCrudden and Schraw, 2007; McCrudden, 2011; Dunlosky et al., 2013): Instead of providing the complete instructional material to learners, with elaborative interrogation learners are prompted by questions to generate parts of the instructional material (cf. Dunlosky et al., 2013). These prompts promote explanatory inferences by asking learners questions that relate to relevant aspects of the contents (McCrudden and Schraw, 2007; McCrudden, 2011). Since generating parts of the contents may be perceived as more difficult than simply reading the contents (Bjork and Bjork, 2011; Navratil and Kühl, 2019), it may reduce overconfidence in the learning success (Berthold and Renkl, 2010; Bjork et al., 2013; Kühl et al., 2018). Moreover, generating parts of the contents requires a higher degree of effortful and analytic processing of information than simply receiving the complete study material. Therefore, it is expected that learners will also better concentrate and invest more mental effort when they need to generate parts of the contents, thereby processing the content more deeply. This in turn is supposed to result in a better comprehension of the learning contents (Craik and Lockhart, 1972).

In a similar way, the potential benefit of elaborative interrogation may also be explained against the backdrop of generative learning theories (cf. Wittrock, 1974; Chi, 2009; Fiorella and Mayer, 2016). Roughly summarized, according to generative learning theories, learners need to make sense of the presented information by actively engaging with the information, so that a meaningful learning to occur. Referring to Fiorella and Mayer (2016); see also Mayer, 2009), this goal can be achieved by generative processes of organizing the information into a coherent mental model and integrating the information with prior knowledge. Elaborative interrogation may require from learners to invest mental effort to actively carry out these generative processes, which in the end should lead to better learning outcomes compared to control conditions that do not have to generate information.

When considering the research about the effectiveness of elaborative interrogation on learning outcomes, there is overall generally a positive effect (cf. Roediger and Pyc, 2012; Dunlosky et al., 2013). Thereby, as pointed out by Dunlosky et al. (2013), in the majority of studies learning outcomes were assessed by factual knowledge questions such as cued recall, matching tasks and recognition tasks. Though, only a few studies assessed open free-recall tests and even fewer studies used measures that reflect a deeper understanding of the content, that are for instance reflected in transfer tests (cf. Dunlosky et al., 2013). However, since we consider these latter measures as especially important in educational contexts, they were in the focus of the current study.

It is conceivable that for elaborative interrogation to be beneficial for learning, learners also need resources available to be able to correctly generate parts of the study material as it usually involves effortful analytic reasoning. In this connection, one potentially important factor is controlled attention and information processing, that is, cognitive self-control (Schmeichel et al., 2003). Such controlled cognitive processes refer to the active, conscious, and relatively effortful cognitive engagement while working on a problem (i.e., the operations of System 2; see the next paragraph). Cognitive control seems to be a limited resource that can be depleted, which will be explicated in the following.

Cognitive control (or self-control) can be conceptualized as being integrated in dual process theories of information processing (Hofmann et al., 2009; Bertrams et al., 2015). A central assumption in these theories is that there are two systems (or types) of information processing (Evans, 2008; Morewedge and Kahneman, 2010): System 1 refers to the automatic, fast, and mostly unconscious and effortless operations of associative memory; it generates impressions, intuitions and response tendencies. In contrast, the operations of System 2 are slower, mostly conscious, and effortful. It monitors, sometimes rejects and sometimes modifies and makes explicit the response tendencies generated by System 1. Higher order thinking processes that require controlled attention and information processing, such as analytic reasoning, are assumed to be dependent on the activation of System 2 (Evans, 2008). In this respect, the posed demands of effortful cognitive processing when learning with elaborative interrogation would require the mobilization of System 2. Bertrams et al. (2015) argued that the mobilization of System 2 may be less likely when individuals’ resources for cognitive control are momentarily depleted compared to being intact. Their rationale was based on theory and research considering self-control as a limited resource (Baumeister et al., 1998; for a review, see Baumeister and Vohs, 2016). Applying a two-task paradigm, it has been demonstrated that people perform lower in subsequent tasks that require self-control when they already had exerted some kind of self-control in an unrelated preceding task compared to a control group without initial self-control demands. A variety of tasks that involve some form of self-control have been used to manipulate as well as to measure self-control. For instance, suppressing any emotional response while watching a humorous video caused less persistence in solving anagrams, a cognitive task that requires to keep up effort despite multiple initial failures (Baumeister et al., 1998). In another study Schmeichel (2007), inhibiting predominant writing tendencies led to lower performance in a working memory task that needed controlled updating of information (reverse digit span task). This pattern was interpreted such that self-control (including controlled information processing) is based on a limited resource akin to strength and can temporarily be depleted (Baumeister et al., 1998; Schmeichel et al., 2003; Schmeichel, 2007). The state of a momentarily depleted self-control resource has been coined *ego depletion* (Baumeister et al., 1998). The processes underlying this observable ego depletion effect are still under discussion (Baumeister and Vohs, 2016), however, the main issue for the present research is that initial self-control demands can cause decrements in controlled cognitive processes associated with System 2 and, thus, impair the momentary ability to reason in an analytical way. There is ample evidence from different labs for the detrimental effect of ego depletion on System 2 operations (e.g., Schmeichel et al., 2003; Masicampo and Baumeister, 2008; Furley et al., 2013; Pohl et al., 2013, however, see Singh and Göritz, 2018 for recent null results). Very recently, two preregistered and highly powered experiments revealed that self-control exercised on a writing task caused reduced attention control, which is a crucial ingredient of controlled information processing such as reasoning (Garrison et al., 2018).

Summing up, compared to learning with the complete study material, learners may rely to an even higher degree to an effortful and analytic processing of information when learning with elaborative interrogation, where parts of the study material have to be generated. Hence, to successfully work with elaborative interrogation, learners need available resources so that an effortful processing can occur. If an adequate processing of information is impaired by means of ego depletion, learners may not be able to correctly generate parts of the study material, also leading to incomplete study material. Hence, under conditions of ego depletion, it is conceivable that learning with elaborative interrogation might be rather disadvantageous compared to providing the complete study material. To conclude, the potential benefits of elaborative interrogation might particularly unfold under nondepleted conditions, but might backfire under depleted conditions. This was investigated in the present study.

The used instructional material was an illustrated science text about how airplanes achieve lift. As learning outcome measures, we assessed a retention test (free recall) as well as a transfer test (problem solving). Furthermore, the instructional material was rated by means of items asking for difficulty, mental effort, concentration and feeling of success.

We expected an interaction between the factors learning with elaborative interrogation and ego depletion for our learning outcome measures (Hypothesis 1a): A positive effect on learning outcomes of elaborative interrogation compared to reading should only be observable when participants were not depleted, but elaborative interrogation should be detrimental to learning under conditions of ego depletion, since in this case learners may not properly generate the requested information with the elaborative interrogation. Moreover, we assumed a general negative effect of ego depletion on learning outcomes (Hypothesis 1b). This detrimental effect of ego depletion should also be reflected in the answers given to the elaborative interrogation prompts (Hypothesis 2). We assessed the subjective measures for rather explorative reasons and whether they would mirror the results of the knowledge test. Similarly, we assumed that learners would only be able to invest more effort and concentration in learning with elaborative interrogation compared to a control condition as long as they were not depleted, but not when they were depleted (Hypothesis 3). Also, we assumed that learning with elaborative interrogation compared to a control condition would be perceived as more difficult and reduce overconfidence (lower feeling of success) in learners (Hypothesis 4a) and that learning with elaborative interrogation would be perceived as more difficult and less successful when learners were depleted compared to nondepleted learners (Hypothesis 4b).

## Materials and Methods

### Participants and Design

Ninety-seven persons (96 university students from a German University; one person indicated that she did not study) participated for course credit or sweets. They were randomly assigned to one of four conditions that resulted from a 2 × 2 between-subject design with ego depletion (yes vs. no) and learning condition (elaborative interrogation vs. control) as independent variables. One person indicated that he took already part in a study with the topic of the current study; these data were omitted from further analyses. Of the remaining 96 persons (*M* = 21.76 years, *SD* = 2.05), 56 were female and 40 were male.

An ethics approval by means of an ethical board was not mandatory, neither by the University’s guidelines nor by national regulations in Germany. Nevertheless, there are ethical guidelines of the German Psychological Society’s (DGPs; 2004, CIII) and the whole conducted experiment followed the rules set by these ethical guidelines. All subjects were aware of taking part in research. Before starting the experiment, each participant received a written informed consent, where they were informed about the possibility of quitting the experiment with no repercussions or disadvantage at any time. All participants signed the informed consent and allowed us to use their collected data anonymously for research purposes.

### Ego Depletion Task

The task to manipulate ego depletion was a writing task that was identical applied in previous research (e.g., Bertrams et al., 2013, 2015) to induce ego depletion: All participants were instructed to transcribe a text (which was presented on a computer screen) on a blank sheet within a limited amount of time. The text was about the history of the city Mannheim (in which the University is located where the present study took place) and the given time for writing was 6 min. Participants in the nondepleted conditions were instructed to transcribe the regular text, whereas participants in the depletion conditions were instructed to omit the letters *e* and *n* wherever they would normally appear in their writing (e.g., in the depletion condition, the word Mannheim had to be written Mahim). Note that the letters *e* and *n* are the two most frequent letters in German, so participants had to suppress their well-learned writing habits in order to perform the task correctly. This procedure was also used as a manipulation for ego depletion in prior work, where it had a detrimental effect on the secondary task (e.g., Bertrams et al., 2015; Dummel and Rummel, 2016; Lindner et al., 2017; Schmeichel, 2007), even though this effect it admittedly not always that straightforward (e.g., Wolff et al., 2019).

### Instructional Material

The instructional material dealt with the topic how airplanes achieve lift. It was adapted from Mautone and Mayer (2001) and modified. At this, the modified version was based on the instructional material used by Eitel and Kühl (2016). The instructional material was distributed among five computerized pages and presented online via the web-based software Unipark ^1^. On the first page, the topic was introduced without providing much information that was relevant for the subsequent test. On the second page, the shape of the wing was described. Based upon that, the airflow speed above and below the wing was explained on page 3. Page 4 dealt with the pressure above and below the wing (as a result of the different airflow speed) and the corresponding uplift. Page 5 was not essential to understand how an airplane achieves lift; it was solely described that a wing needs to be long and robust to lift the weight of an airplane. The first four pages contained each a picture and corresponding text, while the last page solely contained text.

**FIGURE 1:**
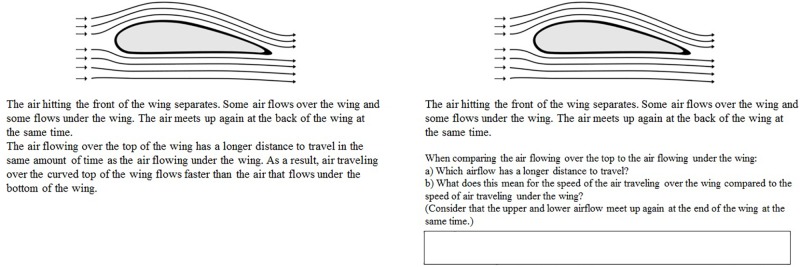
Excerpt of the instructional material. On the left side, the control condition is depicted and on the right side, the elaborative interrogation condition is depicted. Note that the used instructional material was presented in German, but is translated for this figure.

All participants read the same instruction, namely that they would on the following pages learn something about why a plane is flying and that they would afterward receive a knowledge test. For the control condition, the regular text was provided while no prompts were given. The regular text consisted of 327 words (page 1: 53 words; page 2: 39 words; page 3: 84 words; page 4: 92 words; page 5: 59 words). For the elaborative interrogation condition, on page two, three, and four some segments of the regular text were each removed and prompts asking for that particular information were each provided at the bottom of a page. Participants had to type their answers to the questions of the prompts on the computer in a blank field, which was located below the prompt. An example for both conditions is given in Figure 1. Pages one and five, which were not essential for the topic how airplanes achieve lift, were identical with the control condition. The text for the elaborative interrogation conditions contained 209 words (page 1: 53 words; page 2: 14 words; page 3: 42 words; page 4: 41 words; page 5: 59 words). The formulation of the prompts on page 2 contained 32 words, on page 3 it contained 55 words and on page 4 it contained 44 words. Overall, the number of words was comparable between the two learning conditions (327 words or 340 words, respectively). Note that while the instructional material for the control condition was used in the same way in a previous study (Eitel and Kühl, 2016; fluent condition), this was the first time that the elaborative interrogation manipulation for this material was implemented.

Participants controlled the learning environment by pressing a “next”-button to go on to the next page or a “backward”-button to go to the previous page. When participants pressed the “next”-button at the last page of the instructional material, a new page appeared that informed participants that if they press the “next”-button, the learning phase would end. Participants had the opportunity to press a “backward”-button to go back to the instructional material.

### Measures

The measures consisted of a participant questionnaire, a three-item manipulation check concerning the manipulation of ego depletion, answers to the prompts (for the elaborative interrogation conditions), subjective ratings of the learning phase, a knowledge test and a study questionnaire. Moreover, the time participants had spent on each computerized page was logged.

#### Participant Questionnaire

The participant questionnaire asked on the one hand for demographic data (sex, age, field of study, semester of study, final school exam grade, whether German is the mother language and if not, since when the participant speaks German). Moreover, it asked for participants’ self-reported prior knowledge as well as experience with flying. These self-report measures are commonly applied for the used instructional material (cf. Mautone and Mayer, 2001; see also McCrudden et al., 2009; Eitel and Kühl, 2016). Concerning self-rated prior knowledge, participants had to rate their knowledge about the mechanics of flying by two questions [each on a five-point scale ranging from 1 (very low) to 5 (very high)]: “Please rate how much knowledge you have of airplane mechanics.” and “Please rate how much knowledge you have of how airplanes achieve lift.” These two items were summed up to one score (Cronbach’s alpha = 0.83). Moreover, participants had to check mark, whether each of the following six statements would apply for them: “I have flown on a commercial airline”; “I have flown in a noncommercial plane”; “I have sat next to someone while they were flying a plane”; “I have had some instruction to do with aviation”; “I have taken flying lessons”; and “I have flown a plane” (cf. Mautone and Mayer, 2001, p. 380). Participants were awarded one point for each check mark. Surprisingly, three persons indicated that they already have taken flying lessons as well as flown a plane.

#### Manipulation Check

The manipulation check consisted of three items (“How effortful did you find the writing task?,” “How difficult did you find it to execute the writing task?,” and “How much did you suppress your usual writing behavior?”), that were also applied in other studies (e.g., Dummel and Rummel, 2016; Englert and Bertrams, 2016; Lindner et al., 2017). These three items were presented at the computer and had to be rated on a Likert scales ranging from 1 (not at all) to 7 (very). The three items were summed up to one score (Cronbach’s alpha = 0.72)^2^.

#### Students’ Subjective Ratings

Students’ subjective ratings concerning the learning phase were assessed by the items mental effort (“How much mental effort did you invest to understand the contents?”), difficulty (“How difficult was it for you to learn with the material?”), concentration (“How much did you concentrate during learning?”) and feeling of success (“What do you think: How successful will you be in answering a knowledge test?”). These items had to be rated on a seven-point Likert-scale ranging from 1 (not at all) to 7 (very much) and were presented at the computer.

#### Answers to the Elaborative Interrogation

For those participants that received the prompts, the answers to the three elaborative interrogation prompts were scored according to a predefined coding scheme by assessing how many of the core idea units are included in a participant’s answer. Each correctly mentioned core idea unit was awarded one point. The achieved points of the three answers were summed up to one score (maximum score: 7 points). The answers to the elaborative interrogation were scored by two independent raters who were blind to experimental conditions (i.e., with respect to the manipulation of ego depletion). Interrater reliability (two-way random effects, consistency intraclass correlation, average measure) was very good, *ICC* = 0.97. The final score was calculated by the arithmetic mean of the scoring of the raters.

#### Knowledge Test

The knowledge test was adapted from Mautone and Mayer (2001). It consisted of one retention question and four transfer questions. For the retention question, students were asked to write down as much as they can remember about how airplanes achieve lift within 4 min. For each of the four transfer questions, participants had to apply the contents of the instructional material to new problems or scenarios. The four transfer questions were the following (cf. Eitel and Kühl, 2016). (1) “How could an airplane be designed to achieve lift more rapidly?,” (2) “What characteristics of a wing would prevent an airplane from achieving lift?,” (3) “Using what you’ve learned about how airplanes achieve lift, explain how helicopters achieve lift,” (4) “Ailerons are flaps on the rear edge of an airplane wing which the pilot can move up or down. Explain how ailerons affect a plane’s altitude.” For each of the four transfer questions, participants were given 2.5 min.

According to a coding scheme, the retention question was scored by assessing how many of the core idea units are included in a participant’s answer (cf. Mautone and Mayer, 2001; Eitel and Kühl, 2016, pp. 381–382). For instance, students received one point when they wrote that the upper surface of the wing is more curved (or longer); or they received one point when they wrote that the airflow around the upper surface of the wing is faster. Overall, there were nine core idea units (max. score achieved by a student: 6.5 points). For each transfer question, there was also a list of possible correct answers that was adapted and slightly revised from Mautone and Mayer (2001, pp. 381–382). One point was awarded for each correct answer to a transfer questions and the final score for transfer was determined by summing up the points from all four transfer questions. Acceptable answers for transfer questions 1 (how a plane could be redesigned to achieve lift more rapidly) were for instance, (a) to curve the top surface of the wing more (also, increase the surface area of the top of wing, or make the top of the wing longer), (b) to flatten the bottom of the wing more (also, decrease the surface area of the bottom of the wing, or make the bottom of the wing shorter), (c) to increase the air speed over the top of wing, (d) to decrease the airspeed under the bottom of wing, (e) to lower the pressure on the top of the wing, (f) to increase the pressure under the wing, (g) to increase the dispersion of air above the wing, or (h) to concentrate more air under the bottom of the wing. Acceptable responses to the remaining questions followed similar guidelines. For example, some possible acceptable answers for Question 2 (why wing design would prevent a plane from achieving lift) included: (a) to curve the top surface of the wing less (also, decrease the surface area of the top of wing, or make the top of the wing shorter, or (b) to design a wing that is too short). Acceptable responses for the Question 3 (how a helicopter achieves lift) included: (a) the top surface of the blade is more curved or the bottom is flatter than the top, or (b) the air over the top of the blades flows faster than the air under the blades. Some examples of acceptable responses for Question 4 (the effect of aileron position) included: (a) to curve the top surface of the wing more so that the air on the top of the wing moves faster; (b) to curve the top surface of the wing less so that the air on the top of the wing moves slower; (c) to curve the top surface of the wing more so that the pressure on the top of the wing decreases; (d) to curve the top surface of the wing less so that the pressure on the top of the wing decreases; (e) to curve the bottom of the wing more so that the air under the wing moves slower; et cetera. The maximum transfer score that was achieved by a student was 14 points. The retention question and the four transfer questions were each rated by two independent raters who were blind to the experimental conditions. Interrater reliability (two-way random effects, consistency intraclass correlation, average measure) was acceptable for both retention, *ICC* = 0.88, and transfer, *ICC* = 0.86. The final score for each test was calculated by the arithmetic mean of the scoring of the raters.

The knowledge test was not designed to differentiate between concepts that were asked in the prompts and concepts that were described identically in the text of both learning conditions. Rather, the corrects answers for the retention and transfer questions largely addressed what was subject to the manipulation of the learning conditions, meaning that they mainly referred to concepts that were asked by the prompts in the elaborative interrogation condition (and exclusively provided by the text for the control condition, respectively). For instance, eight of the nine core idea units of the retention test were asked by the prompts of the elaborative interrogation conditions. Hence, when analyzing the knowledge test, we did not differentiate between concepts that were asked in the prompts and concepts that were described identically in the text of both learning conditions. Note that even when constructing such differentiated knowledge scores *post hoc*, the pattern of results would not change for the reported analyses below.

#### Study Questionnaire

Participants had to indicate whether they already took part in a study about how airplanes achieve lift (yes or no) and if so, how well they could remember the content (on a five point scale from 1 = very bad to 5 = very good). One participant stated to already have taken part and to remember the contents in a good way (value = 4), and this participant was excluded from data analyses (see section Participants and Design). Participants also had to indicate, whether they already took the writing task in another study (yes or no). At this, 14 participants reported to already have taken the writing task. Moreover, participants were asked whether they had during the study an assumption about the purpose of the study, and if so what they think the purpose was. Also they were asked whether they have any comments about the study. Participants could type their answers to the latter two questions in a blank field on the computer. Eight participants had partly correct assumptions about the purpose of the study concerning the manipulation of ego depletion.

#### Learning Time

Via the web-based software Unipark, the time participants had spent on each computerized page is logged. Thereby, even though it was not in our initial focus, we could retrace and calculate the time participants had spent with the instructional material. It should be noted though, that due to the web-based character, these data may be somewhat imprecise and rather a rough estimate of the time learners actually spend with the instructional material.

### Procedure

The experiment took place in a laboratory at the University of Mannheim, Germany. Before starting the experimental procedure, the experimenter obtained informed consent from each participant. Participants were then seated in cubicles and tested in groups from one to six persons per session. First participants received the participant questionnaire. Thereafter, the task concerning the ego depletion manipulation was given for 6 min, followed by the ego depletion manipulation check. Then participants could start working with the instructional material without any time restriction. When participants ended working with the instructional material, they received the items for the subjective ratings of the learning phase. Then they started with the knowledge test. Participants were asked to wear headphones while completing the paper-based knowledge test. At this, a voice from the computer signaled participants to go to the next task. Additionally, the number of the task they were working on was presented on the computer screen. Participants were told that after the time ended for a task, they had to stop writing. In case participants finished a task before the time ended, they were not allowed to start with the next task. After the knowledge test, participants received the study questionnaire. Thereafter, they received sweets and/or course credit and were thanked for participation. The study took place in the context of a research based project seminar (bachelor course Psychology) and was organized by a group of five psychology students, who recruited participants and conducted the study as instructors.

## Results

Firstly, it was checked whether the manipulation of ego depletion was successful and whether participants possessed similar prerequisites (self-rated prior knowledge and experience with flying) in the four experimental conditions. Thereafter, the dependent variables manipulation check, subjective ratings of the learning phase, learning outcomes, and study time were analyzed by 2 × 2-AN(C)OVAs with the independent variables ego depletion (yes vs. no) and learning condition (elaborative interrogation vs. control condition). The dependent variable performance in answering elaborative interrogation was analyzed by a one-factorial ANCOVA with the independent variable ego depletion, since only participants in the condition elaborative interrogation received these questions. Means and standard deviations of the assessed variables are presented in Table 1.

**Table 1:** Means (and *SD*) as a function of ego depletion and learning condition.

Learning condition	Elaborative interrogation		Control	
Ego depletion	Yes (*n* = 26)	No (*n* = 23)	Yes (*n* = 23)	No (*n* = 24)
**Control Variables**				
Experience with flying	1.69 (1.19)	1.48 (0.59)	1.52 (1.12)	1.38 (0.77)
Self-rated prior knowledge	3.23 (1.48)	2.70 (1.26)	3.48 (1.88)	2.75 (1.45)
Manipulation check	13.00 (3.21)	7.61 (3.24)	12.39 (3.45)	7.96 (2.63)
**Students’ Subjective Ratings**				
Effort	4.46 (1.73)	4.74 (1.48)	3.39 (1.83)	4.25 (1.80)
Difficulty	2.46 (1.45)	3.04 (1.82)	2.30 (1.26)	3.00 (1.44)
Concentration	5.31 (1.26)	4.83 (1.37)	4.74 (1.29)	4.96 (1.20)
Feeling of success	4.46 (1.73)	4.74 (1.48)	3.39 (1.83)	4.25 (1.80)
Answers to elaborative interrogation (max. seven points)	6.06 (0.83)	5.38 (1.71)	NA	NA
**Learning Outcomes**				
Retention	3.74 (1.38)	3.32 (1.44)	3.32 (1.23)	3.35 (1.39)
Transfer	7.44 (2.19)	7.85 (2.57)	7.64 (2.48)	7.09 (3.49)
Learning time (in seconds)	386.67 (142.21)	359.13 (175.53)	118.35 (55.80)	129.67 (42.84)

### Control Variables

For the control variables experience with flying, a two-factorial ANOVA with the independent variables ego depletion and learning condition revealed neither a main effect of ego depletion, *F*(1, 92) = 0.85, *p* = 0.36, nor a main effect of learning condition, *F*(1, 92) = 0.49, *p* = 0.49, nor an interaction, *F*(1, 92) = 0.03, *p* = 0.86. For self-rated prior knowledge concerning how planes fly, a two-factorial ANOVA revealed also no significant main effect of learning condition, *F*(1, 92) = 0.23, *p* = 0.63, and no interaction, *F*(1, 92) = 0.10, *p* = 0.76, but a significant main effect of ego depletion, *F*(1, 92) = 4.07, *p* = 0.046, η^2^_p_ = 0.042, with participants in the ego depletion conditions rating their prior knowledge as higher than learners in the nondepleted conditions. Thus, conditions cannot be considered equal with respect to this potentially important variable. Therefore, self-rated prior knowledge was used as a covariate in the analyses of all dependent variables that are related to the topic of how planes fly (i.e., for all other variables with the exception of the manipulation check). Note that we also controlled for all assessed demographic data, whether conditions could be considered as equal. This was the case.

### Manipulation Check

A two-factorial ANOVA for the score of the manipulation check items revealed no main effect of learning condition, *F*(1, 92) = 0.04, *p* = 0.84, and no interaction, *F*(1, 92) = 0.56, *p* = 0.46. However, participants in the depleted conditions stated that the task was more demanding than participants in the nondepleted conditions, *F*(1, 92) = 58.41, *p* < 0.001, η^2^_p_ = 0.388, indicating that the manipulation worked.

### Subjective Ratings of the Learning Phase

For mental effort, a 2 × 2-ANCOVA revealed no main effect of ego depletion, *F*(1, 91) = 1.76, *p* = 0.19, η^2^_p_ = 0.019, and no interaction, *F*(1, 91) = 0.62, *p* = 0.43, but a main effect of learning condition, with learners in the elaborative interrogation condition stating to have invested more mental effort than learners in the control condition *F*(1, 91) = 4.68 *p* = 0.03, η^2^_p_ = 0.049. This result for effort is not in line with our assumption of an interaction of ego depletion and learning condition (Hypothesis 3). For feeling of success, the 2 × 2-ANCOVA revealed no significant main effect for ego depletion, *F*(1, 91) = 1.73, *p* = 0.19, η^2^_p_ = 0.019, no significant main effect for learning condition, *F*(1, 91) = 1.21, *p* = 0.28, η^2^_p_ = 0.013, and no interaction, *F*(1, 91) = 1.00, *p* = 0.32. For difficulty, the 2 × 2-ANCOVA revealed no effect of learning condition *F*(1, 91) = 0.11, *p* = 0.72 and no interaction, *F*(1, 91) = 0.04, *p* = 0.85, but a main effect of ego depletion, *F*(1, 91) = 4.17, *p* = 0.04, η^2^_p_ = 0.044: Surprisingly, learners in the depleted conditions found learning less difficult than learners in the nondepleted conditions. These results for feeling of success and difficulty contradict our assumptions (Hypotheses 4a and 4b). The covariate was neither significantly related to effort, *F*(1, 91) = 1.67, *p* = 0.20, η^2^_p_ = 0.018, nor to difficulty, *F*(1, 91) = 0.01, *p* = 0.92. However the covariate was significantly related to feeling of success, *F*(1, 91) = 12.15, *p* = 0.001, η^2^_p_ = 0.118.

For the item concentration, we did not conduct an ANCOVA, since the precondition “homogeneity of regression slopes” was violated. To account for this problem, instead of an ANCOVA, we calculated a moderation analysis. Thereby we conducted a multiple linear regression analysis with the centered continuous predictor self-rated prior knowledge and the dichotomous predictors ego depletion and learning condition (which were effect coded), as well as the resulting interaction terms (cf. Aiken and West, 1991). Results revealed neither main effects for self-rated prior knowledge, β = −0.01, *p* = 0.92, nor for ego depletion, β = −0.05, *p* = 0.67, nor for learning condition, β = −0.13, *p* = 0.22, and also no two-way interaction between ego depletion and learning conditions, β = 0.11, *p* = 0.28. Moreover, there was neither an interaction between self-rated prior knowledge and ego depletion, β = 0.06, *p* = 0.60, nor between self-rated prior knowledge and learning condition, β = −0.10, *p* = 0.36. However, a three-way interaction was observable. β = −0.23, *p* = 0.04. Given that no two-way interactions were observable, this three-way interaction was hardly interpretable. Moreover, since this three-way interaction was not in the scope of our research question, we refrain from discussing it any further. Summing up, there was no meaningful influence of experimental conditions on the item concentration^3^.

### Answers to the Elaborative Interrogation

For the dependent variable answers to the elaborative interrogation, only those conditions can be considered that received these prompts during learning (i.e., conditions with elaborative interrogation). We did not conduct an ANCOVA, since the precondition “homogeneity of regression slopes” was violated. To account for this problem, instead of an ANCOVA, we calculated a moderation analysis. For doing this, the SPSS-macro Process v2.16.3 was used (Hayes, 2013) with 5,000 bootstrap samples and the options “mean center for products” and “heteroscedasticity-consistent SEs.” Results revealed no main effect of self-rated prior knowledge, *t* = 1.45, *p* = 0.15, no main effect of ego depletion, *t* = 1.59, *p* = 0.12, but a significant interaction of prior knowledge and ego depletion, *t* = −3.13, *p* = 0.003. Simple slope analyses were used to trace back this interaction. Results revealed that there were no differences between conditions for participants who rated their prior knowledge as high (1 SD above mean), *b* = −0.66, 95% CI [−1.54, 0.23], *t* = −1.49, *p* = 0.14. However, participants performed better in the depleted conditions than in the non-depleted conditions when they rated their prior knowledge as low, *b* = 1.38, 95% CI [0.40, 2.37], *t* = 2.84, *p* = 0.007. Note that for these participants one SD below the mean was replaced with the minimum because one SD below the mean is outside of the range of the data. Please note that since this moderation analysis is mainly conducted due to methodological problems with an ANCOVA, but not derived from our research question, we refrain from (over-)interpreting these results, but solely want to report them^4^.

### Learning Outcomes

With respect to retention, a 2 × 2-ANCOVA showed no effects of ego depletion, *F*(1, 91) = 0.61, *p* = 0.47, no effects of learning condition, *F*(1, 91) = 0.44, *p* = 0.51, and no interaction between ego depletion and learning condition, *F*(1, 91) = 0.66, *p* = 0.42. Similarly, for transfer a 2 × 2-ANCOVA also revealed no main effect of ego depletion, *F*(1, 91) = 0.13, *p* = 0.72, no main effect of learning condition, *F*(1, 91) = 0.20, *p* = 0.66, and no interaction between ego depletion and learning condition, *F*(1, 91) = 0.80, *p* = 0.37. These results are not in line with our main assumption, namely the interaction of ego depletion and learning condition (Hypothesis 1a) and also not in line with the assumption of a general impeding effect of ego depletion (Hypothesis 1b). The covariate was not significantly related to the retention, *F*(1, 91) = 0.26, *p* = 0.61, or to transfer, *F*(1, 91) = 1.36, *p* = 0.25, η^2^_p_ = 0.015.

### Learning Time

A 2 × 2-ANCOVA showed no effects of ego depletion, *F*(1, 91) = 0.17, *p* = 0.69, and no interaction between ego depletion and learning condition, *F*(1, 91) = 0.62, *p* = 0.43, but a significant effect of learning condition, *F*(1, 91) = 103.80, *p* < 0.001, η^2^_p_ = 0.533, with participants receiving elaborative interrogation clearly spending more time with the instructional material than participants in the control condition. The covariate was not significantly related to learning time, *F*(1, 91) = 0.15, *p* = 0.70.

## Discussion

To summarize our main results: Contrary to what we expected (Hypothesis 1a), we did not observe the assumed interaction of learning condition and ego depletion on learning outcomes. Also there was no main effect of learning condition or of ego depletion (Hypothesis 1b) on learning outcomes. Similarly, contrary to Hypotheses 3 and 4, no interaction of ego depletion and learning condition was observable for the subjective ratings of concentration, mental effort, feeling of success and difficulty.

Concerning the factor ego depletion, the manipulation of ego depletion seemed to be successful, as indicated by the manipulation check. However, and contrary to what we supposed in Hypothesis 2, the factor ego depletion did not affect the performance of correctly answering the elaborative interrogation during learning; similarly, ego depletion did not have any impact on learning outcomes. Participants in the nondepleted and depleted conditions were also learning an equally amount of time. Therefore – even though the data of the learning time have to be interpreted cautiously, since they may not be very exact – these learning time data do not speak in favor of the (*post hoc*) assumption that participants in the ego depletion condition may have compensated (or refreshed) by taking more time for learning, which in turn could have served as an explanation for the observed null effect. The factor ego depletion also had no influence on the subjective ratings of effort, concentration and feeling of success. It solely had an influence on the perceived difficulty of the learning content: Surprisingly, participants in the depleted conditions found learning less difficult than participants in the nondepleted conditions. This finding may be interpreted as a kind of “contrast effect” (Kahneman and Miller, 1986), in that the learning conditions were perceived as relatively easier when learners were exposed to the difficult ego depletion task beforehand, which in this case may have served as a reference norm.

With respect to the construct of ego depletion, there is a controversy whether this construct has an impact at all (cf. Carter et al., 2015; Inzlicht et al., 2015; Cunningham and Baumeister, 2016). In a recent attempt to replicate the ego depletion effect across 23 labs, a null effect emerged (Hagger et al., 2016). However, this study has been criticized for methodological flaws (Baumeister and Vohs, 2016; Dang, 2016). Still, there are some other null findings that have been published (e.g., Singh and Göritz, 2018). In contrast, recently, two preregistered and highly powered experiments showed an effect of ego depletion (Garrison et al., 2018). Given the many studies overall that found the ego depletion effect, it is most likely that ego depletion exists but its occurrence seems to depend on moderating conditions. Therefore, we think that the search of moderators concerning ego depletion (or ego depletion as a moderator, respectively) is justified.

Concerning the current study, ego depletion did not have an impact, speaking at first glance for the skeptic view concerning the relevance of ego depletion, at least for cognitive tasks. We used an ego depletion task (writing task) that has shown in previous studies to lead to a decrease in performance in a succeeding demanding cognitive task (e.g., Schmeichel et al., 2003). Therefore, on the one hand our applied ego depletion manipulation can be considered as methodological justified with respect to our research question. On the other hand, it should be noted that in those studies where ego depletion led to a decrease in performance, the cognitive tasks mainly addressed abilities like reasoning tasks or working memory tasks. However, other than in the current study, they were not about understanding a scientific content – which to our knowledge is the first study that investigated ego depletion in learning and understanding complex cause-and-effects chains. Hence, it might be the case that ego depletion would have a significant impact on demanding learning tasks – such as learning with elaborative interrogation – when the intensity of the ego depletion task would be boosted, even though this argumentation was recently challenged (Wolff et al., 2019).

Another factor that may have diminished a potential ego depletion effect is the length of the used learning environment, which was rather short in this experiment (∼300 words). It may be the case that learners might be able to work through a short environment, even when they are slightly depleted. A negative effect of ego depletion may, however, particularly become evident when the learning tasks would be boosted (i.e., a larger learning environment), so that the effect of depletion would emerge. This notion might be investigated in another study. Moreover, it may also be promising to investigate the impact of ego depletion with regard to the generative task of test-taking: While test-taking can lead to beneficial restudy choices (Little and McDaniel, 2015), good choices can be corrupted by ego depletion (e.g., Dummel and Rummel, 2016). Hence, its potential relevance for the framework of desirable difficulties should not be neglected prematurely (cf. Chen et al., 2017).

With respect to learning conditions, we found no positive effect of providing learners with elaborative interrogation compared to providing learners with text on learning outcomes. Also, learning with elaborative interrogation did not reduce overconfidence (i.e., feeling of success), nor did learners concentrate more, nor was it perceived as more difficult. However, learners stated at least to have invested more mental effort when learning with elaborative interrogation compared to the control condition. Likewise, learners spent much more time learning with elaborative interrogation than learners with the complete study material. This is unsurprising, since learners in the elaborative interrogation conditions had to type their answers, which is a time-consuming process. In this respect, one may argue that elaborative interrogation were less efficient compared to the control condition, since learners took more time by similar learning outcomes. Considering the effectiveness, there are several possible reasons for why elaborative interrogation failed to show a positive effect on learning outcomes in this study, whereas it is often reported as a successful strategy in other studies (cf. Roediger and Pyc, 2012; Dunlosky et al., 2013).

First, we used an instructional material that was not used yet in the context of elaborative interrogation, and more importantly, which was rather short. It may be the case and is an open question whether the effectiveness of elaborative interrogation will especially shine through for longer learning environments. Second, one may argue that the elaborative interrogation has been too difficult to answer. This would be problematic, since when participants would have failed to generate the missing information, they would have had an informational disadvantage compared to the participants in the control condition, who received the complete information. However, participants achieved on average above 80% of the possible score when answering elaborative interrogation. Hence, it may be rather assumed that the elaborative interrogation have not been excessively difficult. Nevertheless, it may be the case that when the success rate in answering elaborative interrogation would have been higher, they may have been more beneficial. Somewhat related, it is frequently reported that elaborative interrogation are especially beneficial for learners that already possess a relatively high amount of prior knowledge, but less for learners with rather low prior knowledge (cf. Dunlosky et al., 2013). One possible explanation for this finding may be that prior knowledge permits to adequately work with elaborative interrogation. Given that participants gave roughly 80% correct answers, it is on the one hand unlikely that the missing effect for elaborative interrogation can be solely reduced to a lack of prior knowledge (or more general learning prerequisites). On the other hand, when prior knowledge would be high enough to perfectly answer elaborative interrogation, it may be the case that their potential would completely unfold. Hence, – even though the effects of prior knowledge on answering elaborative interrogation and the relation to learning outcomes is not that straightforward (cf. Dunlosky et al., 2013) – the potential moderating role of prior knowledge might be examined in a future study by using an adequate prior knowledge test. Since the moderating role of prior knowledge was, however, not in the scope of the present study, we did not assess prior knowledge via a test, but simply by a self-report measure, which is commonly applied for this instructional material.

Third, in our knowledge test we used open-ended questions, consisting of a free-recall test (retention) and a transfer test. However, there are surprisingly few studies concerning elaborative interrogation that assessed knowledge by means of open answer formats, and even fewer studies used measures of comprehension and transfer (e.g., McDaniel and Donnelly, 1996; Dornisch and Sperling, 2006; Dornisch et al., 2011; Roelle et al., 2015; Navratil et al., 2018). Overall, the results of these studies concerning the impact of elaborative interrogation on learning outcomes are not that positive, but rather mixed (for an overview see Dunlosky et al., 2013). It may hence be the case (from an empirical point of view) that a positive effect of elaborative interrogation is more difficult to observe when learners have to freely recall or apply their acquired knowledge. This may be worthwhile to investigate systematically in ongoing studies. Fourth, our knowledge test was assessed (almost) immediate after learning. One the one hand, it is fair to say that in most studies the knowledge test was assessed immediately after learning and that hence time of testing cannot per se explain the inconsistency between our findings and the overall positive evaluation of elaborative interrogation. On the other hand – as is pointed out in the context of desirable difficulties – the beneficial effects of conditions that lead learners to a more active engagement with the instructional material may lead to more sustainable knowledge representation that in turn may be best assessed in a delayed test (cf. Roediger and Karpicke, 2006; Bjork and Bjork, 2011; Schweppe and Rummer, 2016). In connection with the abovementioned fact that we used learning outcome measures for which the benefit of elaborative interrogation is less clear, it may be the case that the potential of elaborative interrogation did not unfold in the current study, since we assessed the knowledge test subsequent after learning. Given the promising effect of elaborative interrogation in the few studies that used a delayed test (cf. Dunlosky et al., 2013), even though there are exceptions (e.g., Dornisch et al., 2011), it may be worthwhile in future studies to use a delayed knowledge test for investigating whether elaborative interrogation produces durable gains in learning (cf. Dunlosky et al., 2013).

## Conclusion

To conclude, other than expected, we did not observe an interaction of learning condition and ego depletion on learning success. Even though the impact of ego depletion is recently questioned, we think it would be premature to deny its potential impact on the framework of desirable difficulties (cf. Chen et al., 2017). Moreover, we think that it may be a fruitful avenue for ongoing research to investigate whether learning with elaborative interrogation may especially pay off in the long run when applying learning outcome measures that address a deeper comprehension of the content.

## Author Contributions

TK substantial contributions to conception, acquisition, and interpretation of data for the work, and drafting the work. AB substantial contributions to conception for the work and revising the work critically.

## Conflict of Interest Statement

The authors declare that the research was conducted in the absence of any commercial or financial relationships that could be construed as a potential conflict of interest.

## Footnotes

1 www.unipark.com

2 We did not assess momentary exhaustion with additional items in order to not explicitly influence the participants toward behaving exhausted in the subsequent task. Please also note that the ego depletion literature does not claim that the subjective experience of exhaustion is necessary for the ego depletion effect to be effective.

3 Please note that when solely conducting an ANOVA, the two main effects for ego depletion and learning conditions, as well as the interaction of ego depletion and learning conditions remain non-significant.

4 Please note that when solely conducting an ANOVA, the nonsignificant effect of ego depletion remains.

## References

[B1] Aiken L. S. West S. G. (1991). *Multiple Regression: Testing and Interpreting Interactions.* Newbury Park, CA: Sage.

[B2] Baumeister R. F. Bratslavsky E. Muraven M. Tice D. M. (1998). Ego depletion: is the active self a limited resource? *J. Pers. Soc. Psychol.* 74 1252–1265. 10.1037/0022-3514.74.5.1252 9599441

[B3] Baumeister R. F. Vohs K. D. (2016). Strength model of self-regulation as limited resource: assessment, controversies, update. *Adv. Exp. Soc. Psychol.* 54 67–127. 10.1016/bs.aesp.2016.04.001

[B4] Berthold K. Renkl A. (2010). How to foster active processing of explanations in instructional communication. *Educ. Psychol. Rev.* 22 25–40. 10.1007/s10648-010-9124-9

[B5] Bertrams A. Baumeister R. F. Englert C. Furley P. (2015). Ego depletion in color priming research: self-control strength moderates the detrimental effect of red on cognitive test performance. *Pers. Soc. Psychol. Bull.* 41 311–322. 10.1177/0146167214564968 25567999

[B6] Bertrams A. Englert C. Dickhäuser O. Baumeister R. F. (2013). Role of self-control strength in the relation between anxiety and cognitive performance. *Emotion* 13 668–680. 10.1037/a0031921 23527509

[B7] Bjork E. L. Bjork R. A. (2011). “Making things hard on yourself, but in a good way: creating desirable difficulties to enhance learning,” in *Psychology and the Real world. Essays Illustrating Fundamental Contributions to Society*, eds Gernsbacher M. A. Pew R. W. Hough L. M. (New York, NY: Worth Publishers), 55–64.

[B8] Bjork R. A. (1994). “Memory and metamemory considerations in the training of human beings,” in *Metacognition: Knowing About Knowing*, eds Metcalfe J. Shimamura A. P. (Cambridge, MA: MIT Press), 185–205.

[B9] Bjork R. A. Dunlosky J. Kornell N. (2013). Self-regulated learning: beliefs, techniques, and illusions. *Annu. Rev. Psychol.* 64 417–444. 10.1146/annurev-psych-113011-143823 23020639

[B10] Carter E. C. Kofler L. M. Forster D. E. Mccullough M. E. (2015). A series of meta-analytic tests of the depletion effect: self-control does not seem to rely on a limited resource. *J. Exp. Psychol.* 144 796–815. 10.1037/xge0000083 26076043

[B11] Chen O. Castro-Alonso J. C. Paas F. Sweller J. (2017). Extending cognitive load theory to incorporate working memory resource depletion: evidence from the spacing effect. *Educ. Psychol. Rev.* 30 483–501. 10.1007/s10648-017-9426-2

[B12] Chi M. T. H. (2009). Active-constructive-interactive: a conceptual framework for differentiating learning activities. *Top. Cogn. Sci.* 1 73–105. 10.1111/j.1756-8765.2008.01005.x 25164801

[B13] Craik F. I. M. Lockhart R. S. (1972). Levels of processing: a framework for memory research. *J. Verbal Learn. Verbal Behav.* 11 671–684. 10.1016/S0022-5371(72)80001-X

[B14] Cunningham M. R. Baumeister R. F. (2016). How to make nothing out of something: analyses of the impact of study sampling and statistical interpretation in misleading meta-analytic conclusions. *Front. Psychol.* 7:1639. 10.3389/fpsyg.2016.01639 27826272 PMC5079083

[B15] Dang J. (2016). Commentary: a multilab preregistered replication of the ego-depletion effect. *Front. Psychol.* 7:1155. 10.3389/fpsyg.2016.01155 27535004 PMC4971805

[B16] Dornisch M. Sperling R. A. Zeruth J. A. (2011). The effects of levels of elaboration on learners’ strategic processing of text. *Instr. Sci.* 39 1–26. 10.1007/s11251-009-9111-z

[B17] Dornisch M. M. Sperling R. A. (2006). Facilitating learning from technology-enhanced text: effects of prompted elaborative interrogation. *J. Educ. Res.* 99 156–165. 10.3200/JOER.99.3.156-166

[B18] Dummel S. Rummel J. (2016). Effects of ego-depletion on choice behaviour in a multi-attribute decision task. *J. Cogn. Psychol.* 28 374–383. 10.1080/20445911.2015.1135929

[B19] Dunlosky J. Rawson K. A. Marsh E. J. Nathan M. J. Willingham D. T. (2013). Improving students’ learning with effective learning techniques: promising directions from cognitive and educational psychology. *Psychol. Sci. Public Interest* 14 4–58. 10.1177/1529100612453266 26173288

[B20] Eitel A. Kühl T. (2016). Effects of disfluency and test expectancy on learning with text. *Metacogn. Learn.* 11 107–121. 10.1007/s11409-015-9145-3

[B21] Englert C. Bertrams A. (2016). Worry activation impairs intelligence test performance only under ego depletion. *Swiss J. Psychol.* 75 161–166. 10.1024/1421-0185/a000179

[B22] Evans J. S. (2008). Dual-processing accounts of reasoning, judgment, and social cognition. *Annu. Rev. Psychol.* 59 255–278. 10.1146/annurev.psych.59.103006.093629 18154502

[B23] Fiorella L. Mayer R. E. (2016). Eight ways to promote generative learning. *Educ. Psychol. Rev.* 28 717–741. 10.1007/s10648-015-9348-9

[B24] Furley P. Bertrams A. Englert C. Delphia A. (2013). Ego depletion, attentional control, and decision making in sport. *Psychol. Sport Exerc.* 14 900–904. 10.1016/j.psychsport.2013.08.006

[B25] Garrison K. E. Finley A. J. Schmeichel B. J. (2018). Ego depletion reduces attention control: evidence from two high-powered preregistered experiments. *Pers. Soc. Psychol. Bull.* 10.1177/0146167218796473 [Epub ahead of print]. 30239268

[B26] Hagger M. S. Chatzisarantis N. L. D. Alberts H. Anggono C. O. Batailler C. Birt A. R. (2016). A multilab preregistered replication of the ego-depletion effect. *Perspect. Psychol. Sci.* 11 546–573. 10.1177/1745691616652873 27474142

[B27] Hayes A. F. (2013). *Introduction to Mediation, Moderation, and Conditional Process Analysis: A Regression-based Approach.* New York, NY: Guilford Press.

[B28] Hofmann W. Friese M. Strack F. (2009). Impulse and self-control from a dual-systems perspective. *Perspect. Psychol. Sci.* 4 162–176. 10.1111/j.1745-6924.2009.01116.x 26158943

[B29] Inzlicht M. Gervais W. Berkman E. (2015). *Bias-Correction Techniques Alone Cannot Determine Whether Ego Depletion is Different From Zero: Commentary on Carter, Kofler, Forster, & McCullough, 2015.* 10.2139/ssrn.2659409 (accessed March 20, 2019).

[B30] Kahneman D. Miller D. T. (1986). Norm theory: comparing reality to its alternatives. *Psychol. Rev.* 93 136–153. 10.1080/02699931.2018.1504747 30096994

[B31] Kühl T. Navratil S. D. Münzer S. (2018). Animations and static pictures: the influence of prompting and time of testing. *Learn. Instr.* 58 201–209. 10.1016/j.learninstruc.2018.07.006

[B32] Lindner C. Nagy G. Ramos Arhuis W. A. Retelsdorf J. (2017). A new perspective on the interplay between self-control and cognitive performance: modeling progressive depletion patterns. *PLoS One* 12:e0180149. 10.1371/journal.pone.0180149 28662176 PMC5491132

[B33] Little J. L. McDaniel M. A. (2015). Metamemory monitoring and control following retrieval practice for text. *Mem. Cogn.* 43 85–98. 10.3758/s13421-014-0453-7 25135813

[B34] Masicampo E. J. Baumeister R. F. (2008). Toward a physiology of dual-process reasoning and judgment: lemonade, willpower, and expensive rule-based analysis. *Psychol. Sci.* 19 255–260. 10.1111/j.1467-9280.2008.02077.x 18315798

[B35] Mautone P. D. Mayer R. E. (2001). Signaling as a cognitive guide in multimedia learning. *J. Educ. Psychol.* 93 377–389. 10.1037/0022-0663.93.2.377

[B36] Mayer R. E. (2009). *Multimedia Learning*, 2nd Edn. Cambridge: Cambridge University Press 10.1017/CBO9780511811678

[B37] McCrudden M. T. (2011). Do specific relevance instructions promote transfer appropriate processing? *Instr. Sci.* 39 865–879. 10.1007/s11251-010-9158-x

[B38] McCrudden M. T. Schraw G. (2007). Relevance and goal-focusing in text processing. *Educ. Psychol. Rev.* 19 113–139. 10.1007/s10648-006-9010-7

[B39] McCrudden M. T. Schraw G. Lehman S. (2009). The use of adjunct displays to facilitate comprehension of causal relationships in expository text. *Instr. Sci.* 37 65–86. 10.1007/s11251-007-9036-3

[B40] McDaniel M. A. Donnelly C. M. (1996). Learning with analogy and elaborative interrogation. *J. Educ. Psychol.* 88 508–519. 10.1037/0022-0663.88.3.508

[B41] Morewedge C. K. Kahneman D. (2010). Associative processes in intuitive judgment. *Trends Cogn. Sci.* 14 435–440. 10.1016/j.tics.2010.07.004 20696611 PMC5378157

[B42] Navratil S. D. Kühl T. (2019). Learning with elaborative interrogations and the impact of learners’ emotional states. *J. Comput. Assist. Learn.* 35 218–227. 10.1111/jcal.12324

[B43] Navratil S. D. Kühl T. Heidig S. (2018). Why the cells look like that – the influence of learning with emotional design and elaborative interrogations. *Front. Psychol.* 9:1653 10.3389/fpsyg.2018.01653 PMC6137232 30245656

[B44] Pohl R. F. Erdfelder E. Hilbig B. E. Liebke L. Stahlberg D. (2013). Effort reduction after self-control depletion: the role of cognitive resources in use of simple heuristics. *J. Cogn. Psychol.* 25 267–276. 10.1080/20445911.2012.758101

[B45] Roediger H. L. III Pyc M. A. (2012). Inexpensive techniques to improve education: applying cognitive psychology to enhance educational practice. *J. Appl. Res. Mem. Cogn.* 1 242–248. 10.1016/j.jarmac.2012.09.002

[B46] Roediger H. L. I. Karpicke J. D. (2006). Test-enhanced learning: taking memory tests improves long-term retention. *Psychol. Sci.* 17 249–255. 10.1111/j.1467-9280.2006.01693.x 16507066

[B47] Roelle J. Müller C. Roelle D. Berthold K. (2015). Learning from instructional explanations: effects of prompts based on the active-constructive-interactive framework. *PLoS One* 10:e0124115. 10.1371/journal.pone.0124115 25853629 PMC4390325

[B48] Schmeichel B. J. (2007). Attention control, memory updating, and emotion regulation temporarily reduce the capacity for executive control. *J. Exp. Psychol.* 136 241–255. 10.1037/0096-3445.136.2.241 17500649

[B49] Schmeichel B. J. Vohs K. D. Baumeister R. F. (2003). Intellectual performance and ego depletion: role of the self in logical reasoning and other information processing. *J. Pers. Soc. Psychol.* 85 33–46. 10.1037/0022-3514.85.1.33 12872883

[B50] Schweppe J. Rummer R. (2016). Integrating written text and graphics as a desirable difficulty in long-term multimedia learning. *Comput. Hum. Behav.* 60 131–137. 10.1016/j.chb.2016.02.035

[B51] Singh R. K. Göritz A. S. (2018). Ego depletion does not interfere with working memory performance. *Front. Psychol.* 9:538. 10.3389/fpsyg.2018.00538 29706923 PMC5907684

[B52] Sweller J. Ayres P. Kalyuga S. (eds). (2011). *Cognitive Load Theory.* New York, NY: Springer 10.1007/978-1-4419-8126-4

[B53] Wittrock M. C. (1974). Learning as a generative process. *Educ. Psychol.* 11 87–95. 10.1080/00461520903433554

[B54] Wolff W. Sieber V. Bieleke M. Englert C. (2019). *Task Duration and Task Order Do Not Matter: No Effect on Self-Control Performance.* 10.31234/osf.io/ub4p5 (accessed March 20, 2019). 31321518

